# Artificial neural networks predict the need for permanent cerebrospinal fluid diversion following posterior fossa tumor resection

**DOI:** 10.1093/noajnl/vdac145

**Published:** 2022-09-13

**Authors:** David P Bray, Hassan Saad, James Miller Douglas, Dayton Grogan, Reem A Dawoud, Jocelyn Chow, Christopher Deibert, Gustavo Pradilla, Edjah K Nduom, Jeffrey J Olson, Ali M Alawieh, Kimberly B Hoang

**Affiliations:** Department of Neurosurgery, Emory University School of Medicine, Atlanta, Georgia, USA; Department of Neurosurgery, Emory University School of Medicine, Atlanta, Georgia, USA; Emory School of Medicine, Atlanta, Georgia, USA; Medical College of Georgia-Augusta University, Augusta, Georgia, USA; Emory School of Medicine, Atlanta, Georgia, USA; College of Arts and Sciences, Emory University, Atlanta, Georgia, USA; Department of Neurosurgery, Emory University School of Medicine, Atlanta, Georgia, USA; Department of Neurosurgery, Emory University School of Medicine, Atlanta, Georgia, USA; Department of Neurosurgery, Emory University School of Medicine, Atlanta, Georgia, USA; Department of Neurosurgery, Emory University School of Medicine, Atlanta, Georgia, USA; Department of Neurosurgery, Emory University School of Medicine, Atlanta, Georgia, USA; Department of Neurosurgery, Emory University School of Medicine, Atlanta, Georgia, USA

**Keywords:** artificial neural networks, machine learning, metastasis, posterior fossa tumor, ventriculoperitoneal shunt

## Abstract

**Background:**

Resection of posterior fossa tumors (PFTs) can result in hydrocephalus that requires permanent cerebrospinal fluid (CSF) diversion. Our goal was to prospectively validate a machine-learning model to predict postoperative hydrocephalus after PFT surgery requiring permanent CSF diversion.

**Methods:**

We collected preoperative and postoperative variables on 518 patients that underwent PFT surgery at our center in a retrospective fashion to train several statistical classifiers to predict the need for permanent CSF diversion as a binary class. A total of 62 classifiers relevant to our data structure were surveyed, including regression models, decision trees, Bayesian models, and multilayer perceptron artificial neural networks (ANN). Models were trained using the (*N* = 518) retrospective data using 10-fold cross-validation to obtain accuracy metrics. Given the low incidence of our positive outcome (12%), we used the positive predictive value along with the area under the receiver operating characteristic curve (AUC) to compare models. The best performing model was then prospectively validated on a set of 90 patients.

**Results:**

Twelve percent of patients required permanent CSF diversion after PFT surgery. Of the trained models, 8 classifiers had an AUC greater than 0.5 on prospective testing. ANNs demonstrated the highest AUC of 0.902 with a positive predictive value of 83.3%. Despite comparable AUC, the remaining classifiers had a true positive rate below 35% (compared to ANN, *P* < .0001). The negative predictive value of the ANN model was 98.8%.

**Conclusions:**

ANN-based models can reliably predict the need for ventriculoperitoneal shunt after PFT surgery.

**Key Point**

- Adaptive neural networks predict need for VPS in patients with p-fossa tumors.

**Importance of the Study**

Hydrocephalus is a prevalent complication after resection of PFTs. Clinicians need a durable method to predict the occurrence of CSF diversion in this population. Defining patients at high risk of ventriculoperitoneal shunt (VPS) will decrease hospital stay time and healthcare expenditures. We applied multiple machine-learning algorithms to retrospectively collected data and found that ANNs could be used to best predict need for long-term CSF diversion after PFT resection. We will next develop a web-based application to help clinicians predict which patients will require a VPS. These data will continue to make our models more robust, and therefore more applicable to future patients.

A well-known complication after resection of posterior fossa tumors (PFTs) is postoperative hydrocephalus (HCP).^1–3^ Postoperative HCP can necessitate long-term cerebrospinal fluid (CSF) diversion, which is treated with the ventriculoperitoneal shunt (VPS). Diagnosing persistent HCP and surgery for VPS may increase hospitalization times and the cost of care for patients with PFTs.

Neurosurgeons employ preoperative VPS/endoscopic third ventriculostomy, pre-/postoperative external ventricular drainage (EVD), and avoiding subtotal resection (STR) and intraventricular hemorrhage (IVH) to combat the risk of postoperative HCP.^4,5^ While some studies from single institutions suggest predictors for postoperative HCP after PFT resection, no novel, machine-learning techniques have been used to predict the need for VPS after PFT surgery.^5,6^

Neurosurgeons sorely need a robust predictor for the risk of requiring VPS after resection of PFTs. In another manuscript, our group performed a multivariate analysis on a retrospectively collected database of predictors of requiring VPS after PFT surgery.^7^ With this study, we performed a validation analysis of predictors of VPS with a machine-learning, artificial neural network (ANN) algorithm on prospectively collected patient cohort. The primary goal of this study was to develop an algorithm that can reliably predict the need for VPS after PFT surgery based upon easily collected, patient-level, and perioperative variables.

## Materials and Methods

### Study Design

This is a multi-site study to develop and prospectively validate a predictive model of need for CSF diversion in adult patients undergoing craniotomy for PFTs within the Emory University Healthcare system from January 2006 to March 2021. This system features 2 tertiary/quaternary academic medical centers, one community-based hospital, and one large public/safety-net hospital. Data were collected from the CTORE (CNS Tumor Outcome Registry at Emory) database, a prospectively maintained database of patient outcomes for central nervous system (CNS) tumors treated at the participating sites. Models were developed and trained based on data from 2006 to December 2019 and then validated upon a prospective cohort consisting of consecutive patients undergoing craniotomy for PFTs between January 2020 and March 2021. The study was approved by the institutional review board at Emory University, and an informed consent waiver was obtained.

### Patient Selection

Patients at least 18 years old or older who underwent a craniotomy for PFT resection within the study period were included. Patients were included regardless of tumor type or suboccipital craniotomy approach. Patients with additional supratentorial lesions were included, provided the posterior fossa lesion was the dominant lesion. In patients who had multiple lesions in the posterior fossa, the largest of the lesions was enumerated in the following analyses. Patients received peri- and postoperative corticosteroids (unless major contraindication) for 1–2 weeks postoperatively. All posterior fossa durostomies were closed with the assistance of a collagen-based, synthetic graft. It is our institutional preference to replace the craniotomy flap after dural closure in all PFT cases, given the known association of craniectomy and postoperative CSF leak.^8^ We do not routinely perform endoscopic third ventriculostomy (ETV) for postoperative hydrocephalus if there is no persistent aqueductal stenosis. Therefore, no patients in our cohort had an ETV performed.

### Data Collection and Model Predictors

Data for model training and validation were obtained from the CTORE database and included variables from a primary review of the patient charts, procedure notes, and imaging data and featured in a previously published manuscript.^7^ Patient charts were reviewed for patient demographics, procedures (eg, EVD placement), surgical complications, pathology, need for permanent CSF diversion (eg, VPS placement), length of stay, and patient final disposition. Preoperative imaging was performed within 1 week of surgical intervention, and postoperative magnetic resonance imaging (MRI) was performed within 72 h of surgery. These MRIs were used to evaluate the location and number of lesions, lesion size, presence of ventriculomegaly (with or without transependymal flow), and extent of resection. Mass effect on the fourth ventricle was also assessed (by authors D.P.B., A.M.A, and K.B.H.) using preoperative MRI and categorized into “no mass effect” (patent ventricle with no effacement), “partial effacement” (any mass effect without complete effacement of the ventricle), or “full effacement.” The location of the lesion was dichotomized into extra-axial and intra-axial lesions. Gross total resection was defined as complete resection noted on the postoperative imaging. Patients that had a residual tumor on postoperative imaging were noted to have STR. Surgical complications were defined as ischemic stroke, postoperative hematoma, postoperative edema requiring decompression, infection, CSF leak, and wound complications.

Previously validated predictors included in the surveyed models along with their classification scheme are summarized in Table 1.^7^ All listed variables were included in this study and were present for all included patients. No imputation was performed for missing variables. Patients with absent imaging data were not included in the study.

**Table 1.** Variables Included in Model Training and Validation

Variables	Type	Classes (if applicable)
Age	Numeric	Continuous Variable
Gender	Nominal	1: Male, 2: Female
Race	Nominal	1:White, 2: Black, 3: Hispanic, 4: Other
Location	Nominal	1: Intra-axial, 2: Extra-axial
Size	Numeric	Continuous Variable
Number of Infratentorial lesions	Numeric	Continuous Integer
Number of Supratentorial lesions	Numeric	Continuous Integer
Hydrocephalus	Binary	0: Absent, 1: Present
Transependymal Flow	Binary	0: Absent, 1: Present
Preoperative EVD Placed	Binary	0: No, 1: Yes
Intra-operative/Postoperative EVD	Binary	0: No, 1: Yes
Mass Effect on fourth Ventricle	Nominal	0: None, 1: Partially effaced, 2: Full effaced, 4: Intraventricular
Surgical Complications	Binary	0: No, 1: Yes
Postoperative IVH	Binary	0: No, 1: Yes
Pathology: Benign/Malignant	Nominal	1: Benign, 2: Malignant
Pathology: Primary/Metastatic	Nominal	1: Primary, 2: Metastatic
Outcome: VPS Placed	Binary	0: No, 1: Yes

EVD, external ventricular drainage; IVH, intraventricular hemorrhage; VPS, ventriculoperitoneal shunt.

### Classifier Training and Validation

We designed a multi-layer perceptron (or ANN) model, using the data mining tool, WEKA, that includes all 17 input variables with 2 hidden layers to predict the 2 outcomes of interest: VPS requirement versus no VPS requirement.^9^ A total of 2 hidden layers were included, given that additional layers did not improve model performance during training. The model was trained using the *N* = 518 observations with 10-fold cross-validation at a learning rate of 0.05 with a batch size of 100. Performance at each fold was assessed using the weighted area under the curve (AUC) for both output classes and the true positive rate for the VPS group. To determine the performance of our ANN model compared to a common classifier, we performed similar training for a total of 16 alternative classifiers that are compatible with our input/output attributes. Surveyed classifiers include Bayes Networks, Simple Logistic Regression, Multinomial Logistic Regression, Support Vector Machine, Stochastic Gradient Descent, Voted Perceptron, 1R Classifier, PART Decision List, Decision Table Majority Classifier, Decision Stump Classifier, Hoeffding Tree, Random Forest, Random Trees, REP Trees, Logistic Model Tree, and J48 Decision Trees. All these classifiers were trained using the training set with 10-fold cross-validation. We performed automated parametrization of the different classifier parameters using Auto-WEKA to determine the optimal parameters for each classifier.^10^ Model performance was assessed against the weighted area under the receiver operating characteristic curve (AUC) and the accuracy in predicting the VPS class (positive predictive value [PPV]) given the relatively low proportion of this class in the data. The top 8 performing models with weighted AUC > 0.5 were reported.

Following training, the ANN model was then tested on a prospective cohort of *N* = 90 patients to evaluate model performance on an external dataset, and similar parameters, including weighted AUC and true positive rates were reported.

### Model Implementation

Instructions on model implementation and execution on independent datasets are available along with sample data in the Supplementary Material.

## Results

### Training and Validation Cohorts

A total of 608 patients underwent craniotomy for PFT within the study period, of which 518 patients were included in the training set, and 90 patients were included in the validation set. The 90 patients in the validation set were prospectively collected from January 2020 to March 2021. Table 2 describes the demographic, imaging, procedural, and outcome variables in the two patient populations.

**Table 2.** Description of Features for the Included Patient Populations

Variable*	Training Cohort (*N* = 518)	Validation Cohort (*N* = 90)
Age	51.5 (15)	53 (16)
Gender: Female	297 [57%]	56 [62%]
Race: White	308 [59%]	62 [69%]
Intra-axial Location	289 [56%]	59 [66%]
Size (cm^3^)	16 (18)	20 (18)
Number of Infratentorial lesions	{1}	{1}
Number of Supratentorial lesions	{1}	{1}
Hydrocephalus	151 [29%]	31 [34%]
Transependymal Flow	60 [12%]	18 [20%]
Preoperative EVD Placed	31 [6%]	8 [9%]
Intra-operative/Postoperative EVD	73 [14%]	14 [16%]
Mass Effect on 4th Ventricle		
No Mass Effect	151 [29%]	11 [12%]
Partially Effaced	285 [55%]	65 [72%]
Completely Effaced	61 [12%]	10 [11%]
Intraventricular	13 [3%]	4 [4%]
Surgical Complications	144 [28%]	27 [30%]
Postoperative IVH	74 [14%]	8 [9%]
Pathology: Benign	235 [45%]	40 [44%]
Pathology: Primary	173 [33%]	31 [34%]
Gross Total Resection	334 [64%]	59 [66%]
Outcome: VPS Placed	68 [13%]	12 [13%]

EVD, external ventricular drainage; IVH, intraventricular hemorrhage; VPS, ventriculoperitoneal shunt.

*Variables reported as Mean (SD) for continuos variable, {Median} for nonguassian variables or *N* [%] for categorical variables.

### Performance of ANN Versus Alternative Classifiers

**Figure 1.**
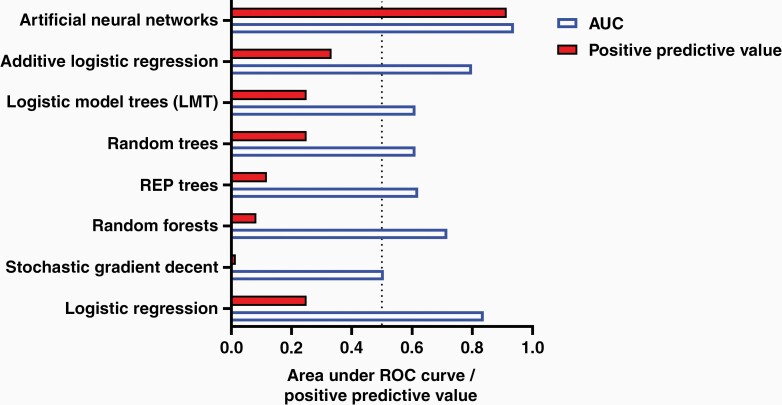
Classifier performance on training dataset using 10-fold cross-validation. Note that while AUC was high for multiple machine-learning based algorithms, the PPV was unacceptably low (less than 0.4) in all cases except for ANNs. ANN, artificial neural networks; PPV, positive predictive value.

The ANN model was trained based on our retrospective data (*N* = 518 patients) using 10-fold cross-validation as described in the methods. Given that the 2 output classes (VPS vs. no VPS) are not balanced (13%), a stratified cross-validation approach was used to ensure balanced class distribution at each fold. Results of cross-validation data are shown in Table 3. A total of 16 alternative classifier types that are compatible with our patient attributes that include a combination of Gaussian distributed, non-Gaussian distributed, and categorical variables were tested to provide comparative analysis. A comparison of our ANN model to the top 7 alternative classifiers is shown in Figure 1.

**Table 3.** ANN Model Performance During Training

Cross-validation Fold (*N*(Train)= 466, *N*(Test)=52)	Weighted AUC	True Positive Rate (VPS Class)
1	0.886	85.70%
2	0.911	85.70%
3	0.999	100%
4	0.886	85.70%
5	0.886	85.70%
6	0.886	85.70%
7	0.999	100%
8	0.927	85.70%
9	0.999	100%
10	0.999	100%
Average	0.938	91.40%

AUC, area under the receiver operating characteristic curve; VPS, ventriculoperitoneal shunt.

Given that 87% of patients in the cohort did not require a VPS, several classifiers had an AUC > 0.5. However, the PPV for these classifiers in predicting the need for VPS in the cohort was low; the highest TP recorded outside of ANN was achieved using additive logistic regression models (AUC = 0.799 and PPV = 33.3%; Figure 1). Using z-score comparison, the performance of ANN was superior to that of additive logistic regression models (*P* < .01).

### External Validation of ANN Model

**Figure 2.**
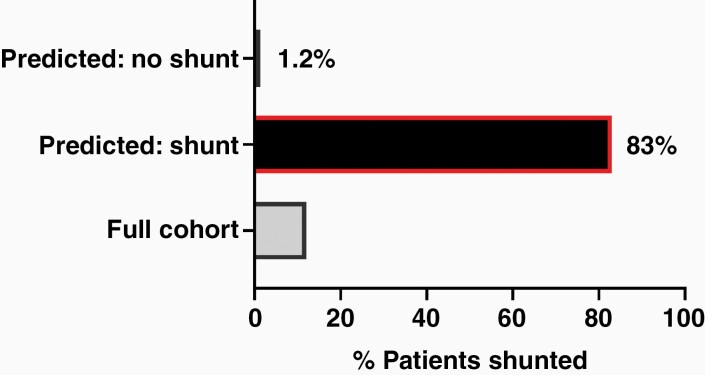
ANN model performance in our prospective cohort per outcome category. The full cohort rate of needing a VPS after PFT resection was 13%. On the prospectively collected data, trained ANNs predicted need for VPS with 83% PPV and predicted no need for shunt (negative predictive value) of 98.8%. ANN, artificial neural networks; AUC, area under the receiver operating characteristic curve; PFT, posterior fossa tumor; PPV, positive predictive value; VPS, ventriculoperitoneal shunt.

We performed external validation of our ANN model on an independent subset of 90 patients prospectively enrolled after model development. Within this dataset, 12 patients (13%) required a VPS, and the overall characteristics of the cohort was similar to those in the training dataset (Table 2). The PPV of our ANN model was 83%, and the negative predictive value was 98.8% (Figure 2).

## Discussion

Our data suggest that the need for permanent CSF diversion is one of the most common complications that occur after craniotomy for PFT, occurring in 13% of cases.^7^ In a previous study, we performed a rigorous descriptive, univariate, and multivariate statistical analysis to identify preoperative and postoperative variables that could be used in machine-learning models.^7^ With this work, we used ANNs to develop a model that predicts, with high accuracy, the subset of patients who will likely require VPS placement postoperatively. The model was validated on an external cohort of prospectively collected patients and implemented in an online resource available for multicenter validation.

VPS is a life-saving procedure for patients with hydrocephalus, but their placement subjects these patients to the additional surgical risk of wound infection, IVH, and shunt failure.^11^ Neurosurgeons attempt to treat hydrocephalus secondary to PFTs temporarily with preoperative EVDs or endoscopic third ventriculostomies; these procedures have their own complications and can subject patients to longer hospitalizations (ie, drain weaning).^4,12^ Others have attempted to devise scoring systems that predict high/low risk for postoperative need for VPS.^2,6^ The scoring systems suffer accuracy as the number of patients in the testing groups is smaller than our cohort, score cut-offs are made somewhat arbitrarily, and model fit was not tested on prospectively collected data. Therefore, the low sensitivity and accuracy of these models make them less helpful for clinical use.

Oncologists, neurosurgeons, and radiation oncologists need a better tool to predict which patients are at high risk of needing VPS after PFT resection such that they can reduce hospital stays and counsel patients/families. Predictive mechanisms could reduce unplanned 30-day readmissions, which may reduce healthcare costs and avoid insurance/governmental penalties.^13,14^ ANN is a computational modeling strategy that can be employed to understand non-linear statistical data for the purposes of clinical decision-making and prediction.^15–17^ Others have used ANNs to predict the risk of cerebral vasospasm after subarachnoid hemorrhage, outcome after traumatic brain injury, or MRI analysis to predict the pathology of different PFTs.^18–20^ To our knowledge, this is the first attempt to use machine-learning-based algorithms to predict the need for VPS after PFT resection.

In this work, we compare the performance of ANN to different alternative classifiers commonly used in clinical data mining. As shown in Figure 1, several classifiers had an overall high AUC; however, the overall accuracy in predicting the need for VPS was low. This is secondary to the low incidence of VPS in the cohort and the need for a more complex model that can predict both classes (VPS and no VPS). For example, a model whose output is always “no VPS required” will correctly classify 87% of the cohort, which will result in an inflated AUC. Careful attention to the accuracy (ie, PPV and negative predictive value) of predicting patients with VPS and without VPS is needed to avoid confirmation bias.

For clinicians that are uninitiated to “black box” machine-learning models, interpretation and evaluation of final models can be disconcerting. In ANNs, no single variable will have a unique overall multiplier or weight. The input features are weighted differently at different nodes within the layers of the network that allows for a unique learning opportunity for trends that are not simply predicted by linear regression models or decision trees. Our data were studied with the optimal rigor of combining both internal validation on the training set and prospective validation on an independent cohort allowing for high confidence in the model’s results.

Logistic regression models performed second best to our ANN model in predicting the need for VPS. These models are commonly used in clinical data analytics with good performance. However, logistic regression models do not account for the complex interactions between different variables; a unique attribute of ANN. ANNs discover hidden layers of nodes, which, in turn, include different combinations of attributes, which increase the degrees of freedom and allow for more robust prediction. Logistic regression models allow for fewer than 20 total degrees of freedom compared to over 100 degrees of freedom that can be attained via ANN using the same variables. From clinical experience, we anticipate significant interactions and inter-dependencies among the study variables (ie, presence of HCP, preoperative EVD placement, tumor size, fourth ventricle mass effect, etc.). These attributes likely provide predictive strength to the overall model that is better captured using ANNs. Indeed, the removal of any single variable from our model resulted in less than a 5% change in overall model performance, further supporting this inter-dependency.

The overall performance of our ANN model was relatively high (83% PPV). Therefore, to ensure there is no over-fitting and confirm the robustness of the model, we first noted the low variability among the different cross-validation folds (Table 3). We additionally performed independent external validation of the model on a new cohort of prospective patients that were not part of the initial training set.

In the future, we will develop a web-based platform such that this model can be used by neurosurgeons around the world. We will employ the prospective data collected through the web-based platform to externally validate the ANN model. These steps will allow for the dissemination of this model to clinical use.

### Limitations

The major limitation of this work is that it is a single-institution study. We have attempted to minimize bias therein with the inclusion of a large number of patients in the cohort, multiple clinical sites, and multiple practicing neurosurgeons. At our institution, we routinely use perioperative corticosteroids, collagen-based, synthetic dural graft for closure, and to avoid craniectomies. Due to the uniformity of these practices at our institution, we did not collect these variables for our analysis. In a multi-institutional database, we will need to include variables concerning the size of craniotomy, craniectomy rate, and dural graft use. To prove model reliability, the ANN model will need to be applied to a multi-institutional database in future studies. Additionally, the ANN model was applied to retrospective data, which has inherent biases. We attempted to ameliorate these biases by external validation of prospectively collected data.
